# Gastroesophageal reflux disease increases the risk of rheumatoid arthritis: a bidirectional two-sample Mendelian randomization study

**DOI:** 10.1038/s41598-024-64966-w

**Published:** 2024-08-01

**Authors:** Quan Yuan, Zixiong Shen, Jiujiang Zhang, Qing Liu, Huimin Whang, Yang Li

**Affiliations:** 1 Department of Thoracic Surgery, Organ Transplantation Center, The First Hospital of Jilin University, Changchun, 130000 China; 2 Department of Cardiovascular Surgery, The First Hospital of Jilin University, Changchun, 130000 China; 3 Department of Dermatology, The First Hospital of Jilin University, Changchun, 130000 China

**Keywords:** Gastroesophageal reflux disease, Rheumatoid arthritis, Mendelian randomization, Bidirectional, Causal effect, Gastroenterology, Medical research, Rheumatology, Risk factors

## Abstract

Rheumatoid arthritis (RA) is a common autoimmune disease, and some observational studies have indicated an association between Gastroesophageal Reflux Disease (GERD) and RA. However, the causal relationship between the two remains uncertain. We used Mendelian randomization (MR) to assess the causal relationship between GERD and RA. Two-sample Mendelian randomization analysis was performed using pooled data from large-scale genome-wide association studies. In addition, we performed multivariate MR analyses to exclude confounding factors between GERD and RA, including smoking quantity, drinking frequency, BMI, depression, and education attainment. The MR results for GERD on RA suggested a causal effect of the genetic susceptibility of GERD on RA (discovery dataset, IVW, odds ratio [OR] = 1.41, 95% confidence interval [CI] 1.22–1.63, *p* = 2.81 × 10^−6^; validation dataset, IVW, OR = 1.38, 95% CI 1.23–1.55, *P* = 1.76 × 10^−8^). Multivariate MR analysis also supports this result. But the results of the reverse MR analysis did not reveal compelling evidence that RA can increase the risk of developing GERD. Our bidirectional Two-Sample Mendelian randomization analysis and multivariate MR analysis provide support for the causal effect of GERD on RA. This discovery could offer new insights for the prevention and treatment of RA.

## Introduction

Gastroesophageal reflux disease (GERD) is a prevalent disorder of the digestive system. Stomach acid and gastric contents flowing back into the esophagus can lead to damage to the esophageal mucosa, resulting in symptoms including but not limited to reflux, heartburn, abdominal pain, chest pain, and difficulty swallowing^1^.

Rheumatoid arthritis (RA) is a chronic inflammation and an autoimmune disease that primarily affects the synovial membranes of joints. It causes pain and swelling in the little limb joints, ultimately resulting in bone erosion and joint deformity. Some RA patients may also experience various organ damage, including the skin, eyes, cardiovascular system, and kidneys. The digestive system can also be directly affected, and gastrointestinal issues in RA patients are often attributed to long-term use of NSAIDs and corticosteroid medications^2^.At the same time, Gastrointestinal symptoms are also one of the major comorbidities contributing to decreased quality of life and increased mortality risk among RA patients^3^.

A study conducted in Japan revealed a significantly elevated risk of GERD among RA patients in comparison to the general population (24.6% vs 11.5%, *p* < 0.001)^4^. A nested case–control study from Taiwan suggested that GERD is an independent risk factor for the occurrence of RA. The risk of developing RA was 2.84 times higher in individuals with GERD compared to the control group (95% CI 2.09–3.85). They performed sensitivity analysis to assess potential bias and after data adjustments, the hazard ratio (HR) remained close to the initial findings at 2.81 (95% CI 2.06–3.83)^5^. A study from South Korea indicated that the adjusted HR for the risk of GERD patients developing RA was 1.49 times higher than the control group (95% CI = 1.37–1.62, *P* < 0.001). Additionally, the risk of RA patients developing GERD was 1.46 times higher than that of the control group (95% CI = 1.38–1.55, *P* < 0.001)^6^. The results of these observational studies suggest a relationship, but not causation, between GERD and RA.

Due to the inherent limitations of observational studies, which cannot fully control for confounding factors and are susceptible to reverse causality and sample size constraints, causal inferences are prone to bias, thus reducing our comprehension of the causal relationship between GERD and RA. Mendelian randomization (MR) is an approach that utilizes genetic variation to assess the presence of a consistent causal effect between an exposure factor and an outcome. Mendelian randomization is also referred to as "nature's randomized controlled trial." An individual's genotype is determined at conception, and genetic variants adhere to Mendel's Second Law, undergoing random allocation during meiosis. Therefore, individuals being naturally predisposed to carry or not carry certain genetic variants associated with a specific risk factor from birth. MR helps to avoid the influence of confounding factors in observational studies and addresses reverse causation, thereby minimizing bias to the greatest extent possible. Some MR studies have shown a causal link between factors such as smoking^7–9^, alcohol consumption^8,9^, depression^10,11^, and educational attainment^12,13^ and GERD or RA. At the same time, some observational studies have also shown that there is a certain association between BMI and them^14,15^. Therefore, we used multivariate MR analysis to adjust for the effects of these factors on our analysis.

We collected publicly available data from large-scale genome-wide association studies (GWAS) and employed a two-sample bidirectional Mendelian randomization approach to analyze and establish the potential bidirectional causal effect between GERD and RA. In addition, we further clarify the reliability of our conclusions by correcting factors such as smoking quantity, drinking frequency, BMI, depression, and education attainment. The results of multivariate adjustment were displayed by IVW and MR-Egger, and Cochrane’s Q value and MR-Egger regression intercept were used to evaluate heterogeneity and pleiotropy, respectively.

## Materials and methods

### Experimental design

**Figure 1:**
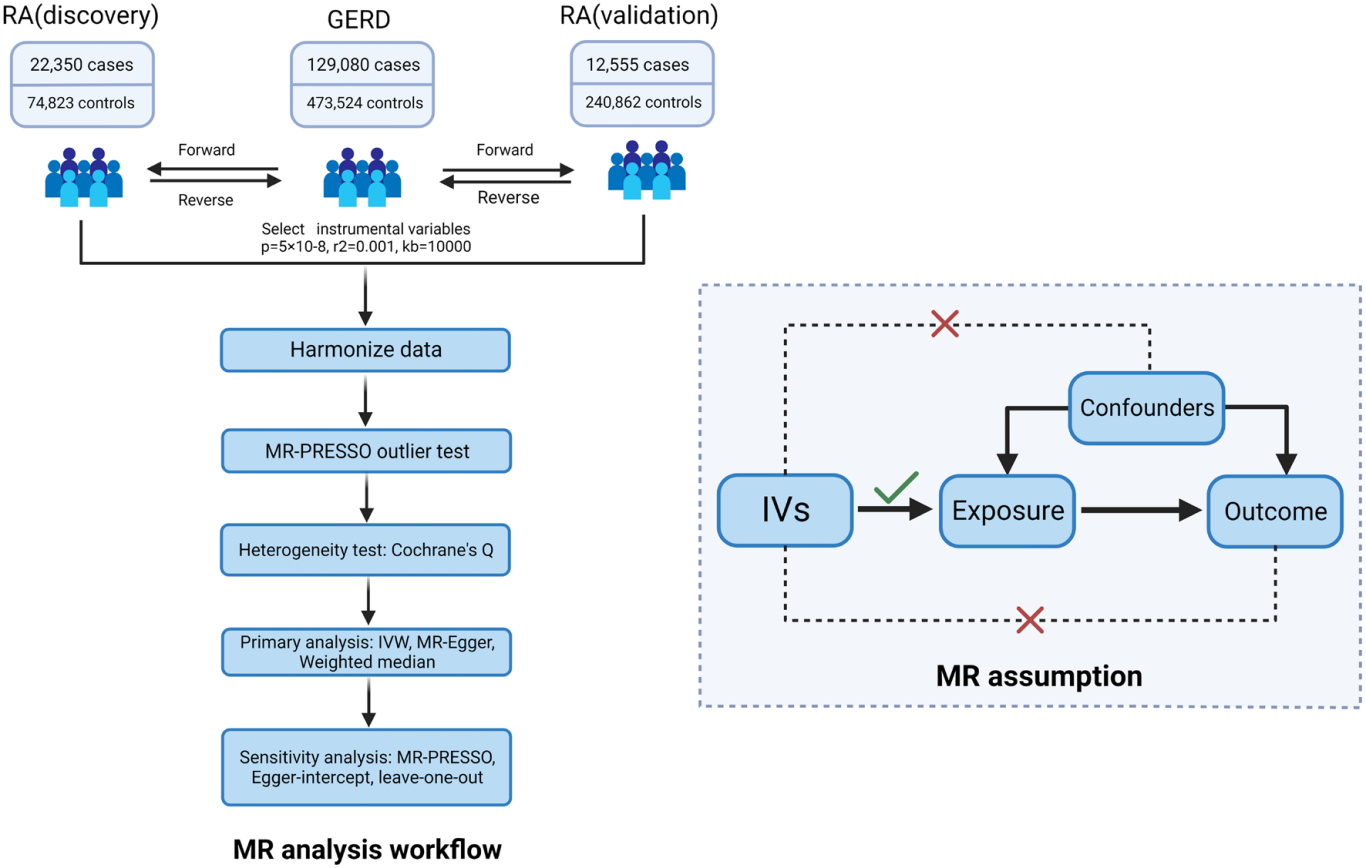
MR analysis workflow and MR assumption. A concise experimental flowchart and the three assumptions that Mendelian randomization analysis needs to fulfill.

Bidirectional two-sample Mendelian randomization (TSMR) analysis must adhere to three essential assumptions: (1) Instrumental variables (IVs) should be highly correlated with the exposure factor; (2) IVs must be independent of confounding variables; (3) IVs cannot affect the outcome through pathways other than the exposure factor (Fig. 1).

## Data sources

### GWAS data for exposure and outcome

We utilized several GWAS datasets for conducting this study. The GERD data were sourced from IEU Open GWAS (https://gwas.mrcieu.ac.uk/), encompassing a total of 602,604 individuals of European ancestry, with the identifier "ebi-a-GCST90000514"^16^. The RA discovery and validation datasets were derived from the GWAS Catalog^17^ (https://www.ebi.ac.uk/gwas) and the FINNGEN database^18^ (https://www.finngen.fi/en), respectively. The former dataset includes individuals of European, East Asian, African, South Asian, and Arab descent, and we specifically utilized the European data. These European data originated from Canada, the Netherlands, Sweden, the United States, the United Kingdom, France, Germany, and Spain, comprising 22,350 cases of European descent and 74,823 European descent control cases. The latter dataset primarily comes from Finland and includes 12,555 cases of European descent and 240,862 European descent control cases (Table 1). All GWAS data used in this study have previously obtained ethical approval from previous research, eliminating the need for additional ethical clearance for the current study. The authors and references can be found in the supplementary tables for further consultation.

**Table 1:** Descriptive details about exposure dataset and outcome dataset.

Exposure or outcome	n cases	Controls	Ancestry	Origin	References or cohort study	PMID	Web source
Gastroesophageal reflux disease	129,080	473,524	EUR	United Kingdom	Ong, J.S. et al. (2022)^16^	34,187,846	https://gwas.mrcieu.ac.uk
Rheumatoid arthritis	22,350	74,823	EUR	Canada North America Netherland Sweden German United Kingdom Spain France	Ishigaki, K. et al. (2022) (discovery)^17^	36,333,501	https://pubmed.ncbi.nlm.nih.gov
	12,555	240,862	EUR	Finland	Kurki, M.I. et al. (2023) (validation)^18^	36,653,562	https://www.finngen.fi/en

### GWAS data for multivariable MR analysis

When analyzing the causal relationship between GERD and RA, we adjusted for the effect of potential confounders on outcomes. We further performed multivariate MR analysis on smoking quantity, drinking frequency, BMI, depression, and education attainment. The datasets of smoking quantity and drinking frequency are cigarettes per day (n = 784,353) and drinks per week (n = 2,965,643) which from the Sequencing Consortium of Alcohol and Nicotine use (GSCAN) project^19^. More information on the study can be found in https://conservancy.umn.edu/handle/11299/241912. The dataset of BMI is from the Genetic Investigation of Anthropometric Traits (GIANT) consortium^20^. The consortium provides meta-analysis data for approximately 700,000 European populations (https://pubmed.ncbi.nlm.nih.gov/30124842/). The depression dataset comes from FINNGEN database^18^, which includes 43,280 cases and 329,192 control cases (https://www.finngen.fi/en). The education attainment GWAS aggregate statistics were derived from the Social Science Genetic Association Consortium (SSGAC), which encompasses education attainment GWAS data, the largest sample size to date, including a total of 71 discovery cohorts including 1,131,881 participants of European ancestry, measuring approximately 10 million SNP loci^21^.

### IVs selection

We used single nucleotide polymorphisms (SNPs) as IVs. To avoid linkage disequilibrium, we established that SNPs associated with the exposure factor must meet the criteria of r^2^ < 0.001, aggregation window = 10,000 kb, significance threshold of *P* < 5.0 × 10^−8^. Subsequently, we extracted SNPs significantly associated with the exposure factor from the outcome data and documented the corresponding allele variants in the supplementary materials. This documentation also includes *p*-values, standard errors, and effect sizes (beta). The F-statistic for each individual SNP was determined using $F=\frac{{beta}^{2}}{{se}^{2}}$ to assess the statistical strength of each IV^22^. The overall F-statistic for SNPs was calculated using $F=\frac{N-K-1}{K}\times \frac{{R}^{2}}{1-{R}^{2}}$, ${R}^{2}$ represents the proportion of variance explained by each SNP, and its calculation formula is ${R}^{2}=2\times eaf\times (1-eaf)\times {beta}^{2}$, where $eaf$ is the effect allele frequency, $N$ represents the sample size of the exposure data, beta denotes the effect of the SNP on the exposure, and $K$ is the number of SNPs^23,24^. $F>10$ indicates a stable association between the SNP and the phenotype, suggesting the absence of weak instrumental variable bias^25^. In addition, proxy SNPs were not utilized in this experiment.

### Statistical analysis

We conducted a bidirectional TSMR analysis on the GERD and RA datasets. We aggregated and harmonized the data to ensure that the SNPs for both the exposure and outcome correspond to the same alleles. We conducted analysis using MR-Egger regression, inverse variance weighting (IVW), weighted median, and Mendelian randomization pleiotropy residual sum and outlier (MR-PRESSO). Simple mode and weighted mode were employed for supplementary result analysis. If all genetic variants meet the IVS assumptions, IVW is used to assess the consistency of the causal effect between exposure and outcome. Cochrane's Q is utilized for assessing heterogeneity. If the *p* > 0.05, we opt for a fixed-effects model; otherwise, a random-effects model is applied. The difference between the intercept of the MR-Egger regression and 0 is employed for an initial evaluation of potential horizontal pleiotropy in IVs. If the *p*-value of its intercept is greater than 0.05, it suggests the absence of horizontal pleiotropy^26^. Sensitivity analysis was conducted using MR-PRESSO test. The MR-PRESSO NbDistribution was set at 3000, and the SignifThreshold = 0.05. MR-PRESSO can also reevaluate pleiotropy by excluding palindromic sequences^27^. If instrumental variables (IVs) show no horizontal pleiotropy, the IVW method is considered the most reliable^28^. In this case, IVW will serve as the primary approach to evaluate the causal effect between GERD and RA. The leave-one-out method is used to determine whether there are individual SNPs that significantly affect the estimation of the causal relationship. Given that there is a causal relationship between GERD and RA in univariate analysis, we further performed multivariate MR analysis to correct for the interference of confounding factors in their causal relationship.

All analyses were conducted using RStudio 4.2.1 (https://www.r-project.org/). The R packages "TwoSampleMR”, "Mendelian randomization", "MVMR", and "MR-PRESSO" were used for MR analysis. Finally, we utilized a web-based tool (https://shiny.cnsgenomics.com/mRnd/) to calculate the MR statistical power^29^. Statistical power is the probability of correctly rejecting the null hypothesis in a hypothesis test, meaning it reflects the ability to detect an actual effect. An appropriate level of statistical power (usually set at 80% or 0.80) ensures that the study is sensitive enough to detect actual effects, reducing the risk of committing a Type II error^30^.

### Ethics approval and consent to participate

Not applicable since the study is based on summary-level data. In all original studies, ethical approval, and consent to participate had been obtained.

## Results

### Univariable MR analysis

We conducted a MR analysis with GERD as the exposure and RA as the outcome. After screening 80 SNPs in GERD, and harmonizing with RA (discovery), a total of 80 SNPs were selected, including 14 palindrome sequences, 3 outliers (rs4713692, rs6722661, rs7942368) and 1 SNPs (rs3828917) strongly associated with the outcome (This SNP was associated with exposure with a *P* value of 2.27 × 10^−8^ but an outcome P value of 6.87 × 10^−18^, violating the three major Mendelian assumptions of randomization, so we manually excluded it. For details, refer to Supplementary Materials 1–2). After harmonization with RA (validation), 78 SNPs were selected, including 3 palindrome sequences,1 outlier (rs4713692) and 1 SNPs (rs3828917) strongly associated with the outcome. (This SNP was associated with exposure with a *P* value of 2.27 × 10^−8^ but an outcome *P* value of 6.19 × 10^–10^, so we also manually excluded it. For details, refer to Supplementary Materials 3–4). After removing these palindrome sequences and SNPs strongly associated with the outcome, we ended up with 62 and 73 SNPs as IVs for the discovery and validation sets, respectively. The F-statistic for all individual SNPs in both subsets were greater than 10. The total R^2^ for the discovery set was 2.82%, with a total F-statistic of 282.43. For the validation set, the total R^2^ was 3.29%, with a total F-statistic of 280.41. The outlier test results from MR-PRESSO indicate that there are 4 outliers in the discovery set (rs4713692, rs6722661, rs7942368, rs3828917) and 2 outliers in the validation set (rs3828917, rs4713692). These outliers have already been included in the previous set of palindrome sequences and SNPs strongly associated with the outcome, and therefore have been excluded accordingly. All outliers can be found in Supplementary Table 9.

**Figure 2:**
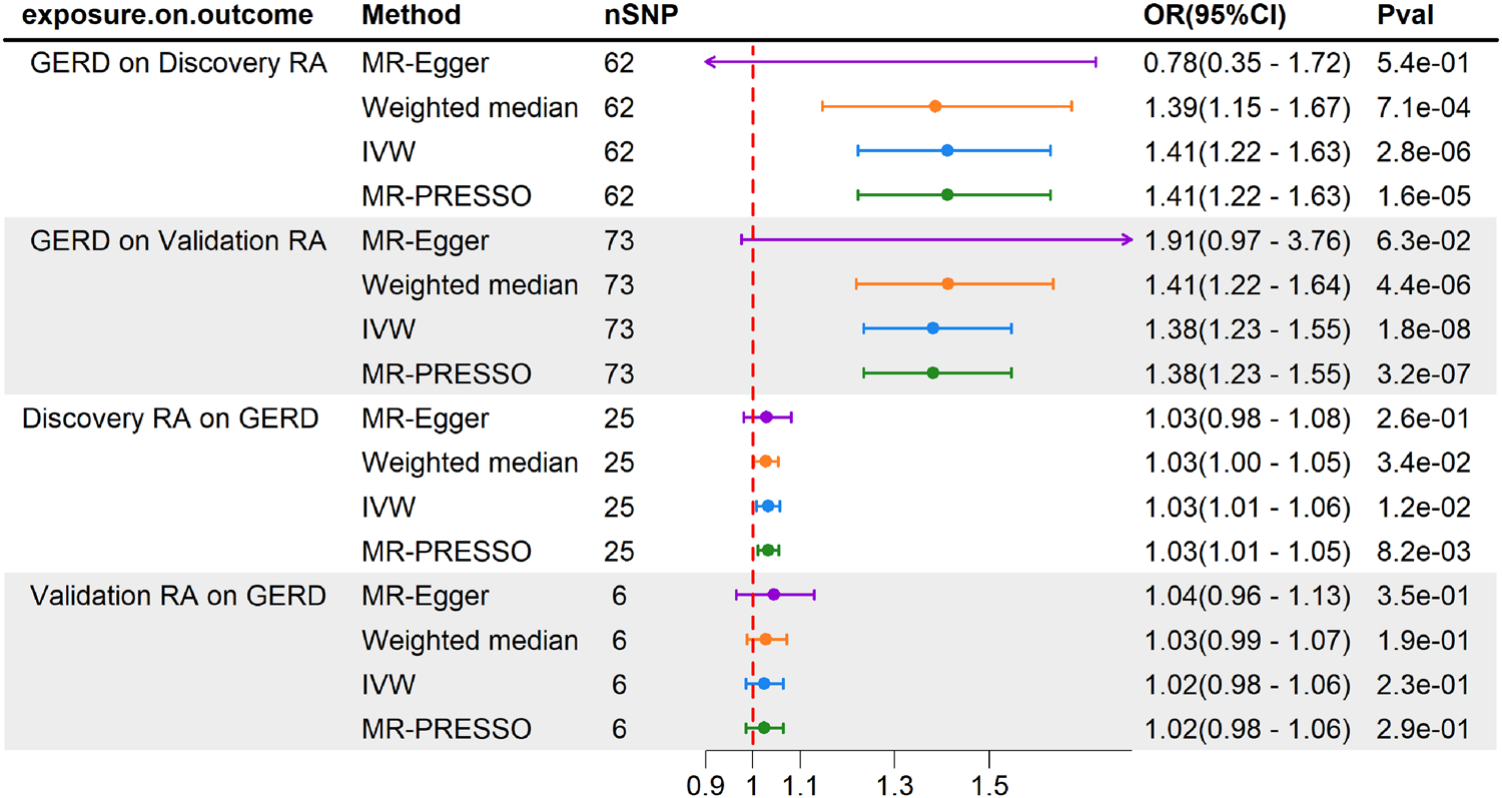
Casual estimate and sensitivity analysis of univariate MR analysis. Causal estimate and sensitivity analysis of MR analysis. Number of SNPs; OR odds ratio; GERD, Gastroesophageal reflux disease; RA, rheumatoid arthritis.

The MR-Egger results indicate that the intercept for the discovery set is 0.020 with *p* = 0.14, while for the validation set, the intercept is − 0.011 with *p* = 0.34. This result suggests the absence of horizontal pleiotropy in the instrumental variables. Therefore, we will consider IVW as the primary method for assessing the causal relationship. The results from IVW suggest a causal effect of the genetic susceptibility of GERD on RA (discovery, OR = 1.41, 95%CI 1.22–1.63, *p* = 2.81 × 10^−6^; validation, OR = 1.38, 95%CI 1.23–1.55, *P* = 1.76 × 10^−8^). The weighted median also supports this causal relationship (discovery, OR = 1.39,95%CI 1.15–1.67, *p* = 7.07 × 10^−4^; Validation, OR = 1.41, 95%CI 1.22–1.64, P = 4.43 × 10^−6^) (Fig. 2). We used the web-based tools mRnd to calculate the statistical power of the MR analysis of GERD to RA (discovery) and RA (validation) to be 100% respectively (Supplementary Table 10).

Next, we conducted a reverse MR analysis with RA as the exposure and GERD as the outcome. Using the previously applied screening criteria, we identified 76 SNPs in RA (discovery), and after harmonizing with GERD, we selected 28 SNPs, including 1 palindrome sequence (For details, refer to Supplementary Materials 5–6). In RA (validation), 24 SNPs were identified, and after harmonization with GERD, 8 SNPs were selected, with no palindrome sequences found (For details, refer to Supplementary Materials 7–8). MR-PRESSO's outlier test results revealed 2 outliers in the discovery set (rs2561477, rs4239702) and 2 outliers in the validation set (rs11758148, rs6065926). After removing palindrome sequences and outliers, we proceeded with the subsequent MR analysis. Ultimately, we obtained 25 and 6 SNPs as IVs for the discovery and validation sets, respectively. The *F*-statistic for all individual SNPs in both subsets were greater than 10. The R^2^ for the discovery set was 15.4%, with a total F-statistic of 710.01. For the validation set, the R^2^ was 4.90%, with a total F-statistic of 2187.70.

The results of the reverse analysis indicate a causal relationship between RA (discovery) and an increased risk of developing GERD (IVW, OR = 1.03, 95% CI 1.00–1.06, *P* = 0.012). However, in the validation set, RA does not show a causal relationship with GERD (IVW, OR = 1.02, 95% CI 0.98–1.06, *P* = 0.235). The results from MR-Egger, weighted median, and MR-PRESSO in both the discovery and validation sets also support their respective conclusions.

### Sensitivity analysis

**Figure 3:**
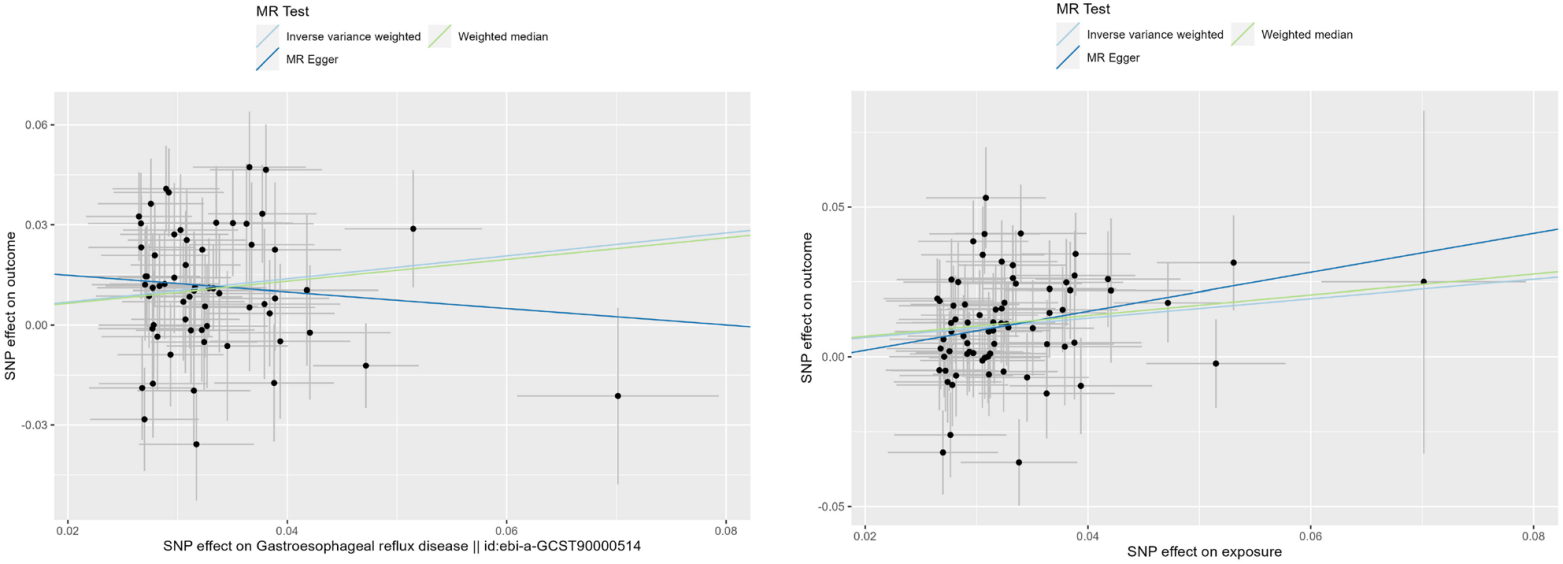
Scatterplot for three methods (GERD on RA). Scatter plots results of MR analysis of GERD as exposure. On the left is GERD on RA discovery, on the right is GERD on RA validation. GERD, Gastroesophageal reflux disease; RA, rheumatoid arthritis.

**Figure 4:**
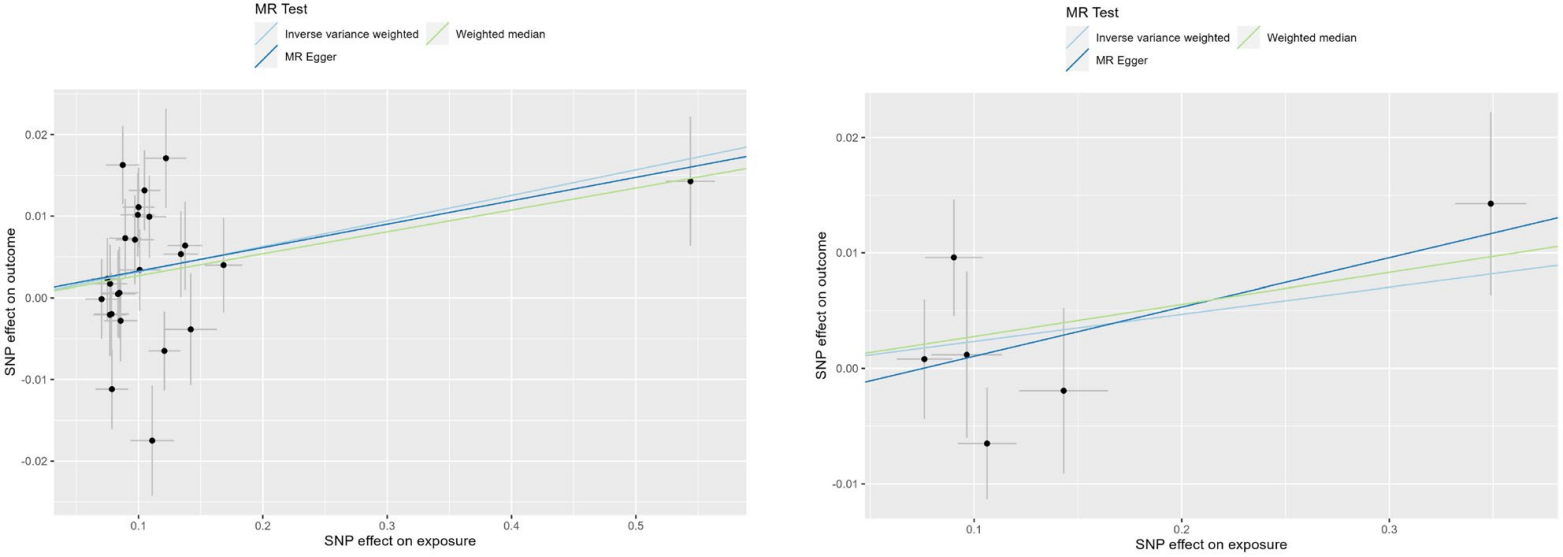
Scatterplot for three methods (RA on GERD). Scatter plots results of MR analysis of RA as exposure. On the left is RA discovery on GERD, on the right is RA validation on GERD.

We conducted heterogeneity analysis using Cochran's Q test for IVs. In the forward MR analysis, we observed heterogeneity in the IVs of the discovery set. Therefore, we employed a random-effects IVW model to assess the causal relationship, and the Egger intercept showed no significant difference from zero. The leave-one-out analysis did not identify any single SNP significantly influencing the causal estimate (discovery, IVW Cochran’s Q = 85.57, *p* = 0.021, MR-Egger Cochran’s Q = 82.52, *p* = 0.029; validation, IVW Cochran’s Q = 87.04, *p* = 0.109, MR-Egger Cochran’s Q = 85.92, p = 0.110). Similarly, in the reverse MR analysis, we found heterogeneity in the IVs of the discovery set, leading us to use the random-effects IVW model for the causal evaluation (discovery, IVW Cochran’s Q = 51.54, *p* = 8.98 × 10^−4^, MR-Egger Cochran’s Q = 51.51, *p* = 5.82 × 10^−4^; validation, IVW Cochran’s Q = 6.86, *p* = 0.231, MR-Egger Cochran’s Q = 6.37, *p* = 0.173) (Table 2).The results from MR-PRESSO support a causal relationship between the genetic susceptibility to GERD and RA. However, the findings differ between the discovery and validation sets regarding whether RA can increase the risk of developing GERD. The scatter plots for both analyses are illustrated in Fig. 3, 4, and the funnel plot and leave-one-out plot can be found in the supplementary materials (supplementary Figures 1–4).

**Table 2:** Heterogeneity results of MR analysis. Total of *F*-statistic and R^2^.

exposure on outcome	IVW			MR-Egger			Total R^2^ (%)	Total *F*-statistic
	Cochran’s Q	Cochran’s Q *P* value	*P* value	Cochran’s Q	Cochran’s Q *P* value	*P* value		
GERD on RA (discovery)	85.57	2.10e^−2^	2.8e^−6^	82.52	2.90e^−2^	5.4e^−1^	2.82	282.43
GERD on RA (validation)	87.04	1.09e^−1^	1.8e^−8^	85.92	1.10e^−1^	6.3e^−2^	3.29	280.41
RA(discovery) on GERD	51.54	8.98e^−4^	1.2e^−2^	51.51	5.82e^−4^	2.6e^−1^	15.40	710.01
RA(validation) on GERD	6.86	2.31e^−1^	2.3e^−1^	6.37	1.73e^−1^	3.5e^−1^	4.90	2187.7

## 3Multivariable MR estimates

**Figure 5:**
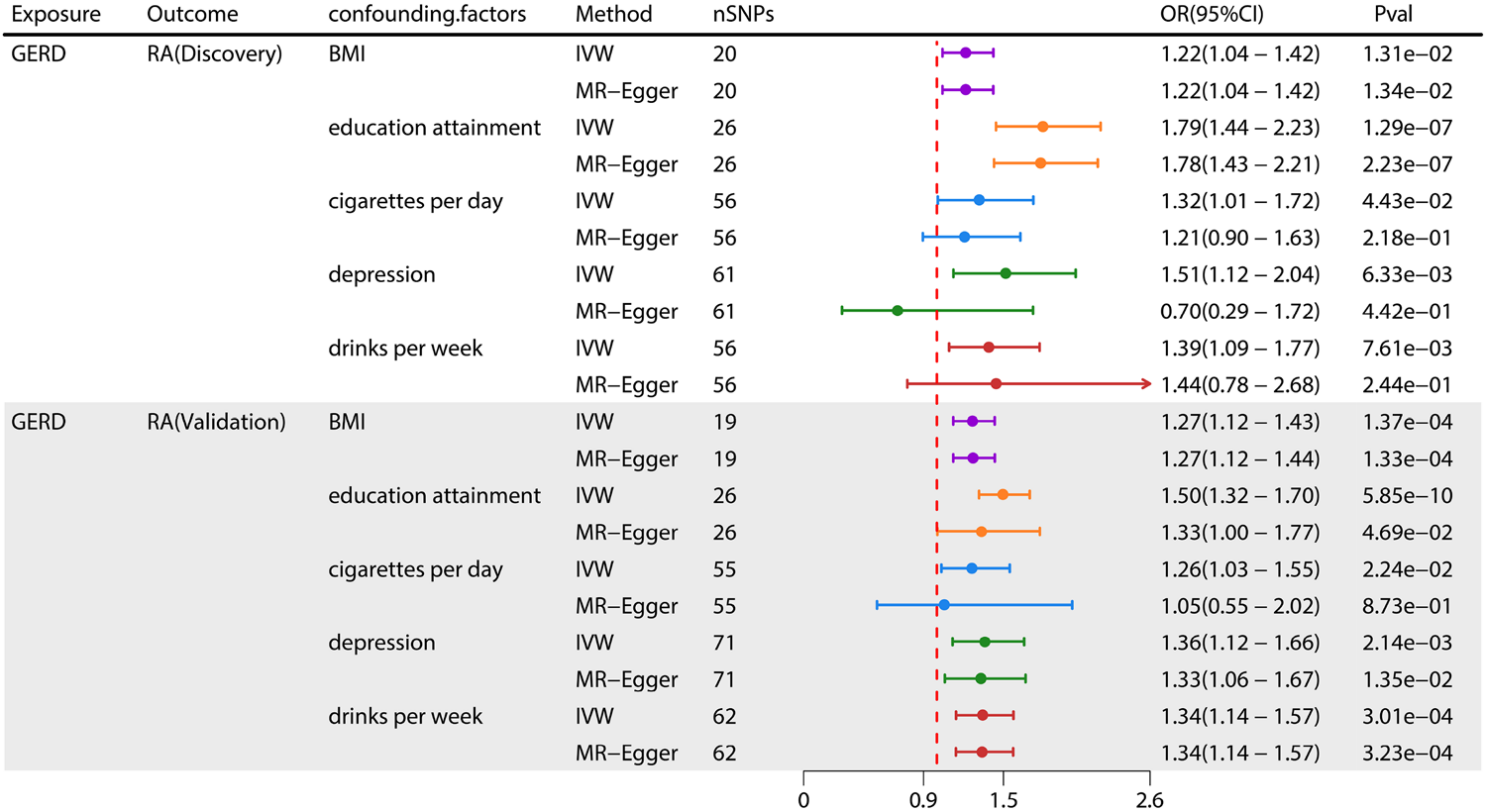
Casual estimate and sensitivity analysis of multivariate MR analysis. Causal estimate analysis of multivariate MR analysis. Number of SNPs; OR odds ratio; GERD, Gastroesophageal reflux disease; RA, rheumatoid arthritis.

For the causal effect of GERD on RA, we performed a multivariate MR analysis to correct for possible confounding factors brought about by smoking quantity, drinking frequency, BMI, depression, and education attainment on RA discovery and validation datasets. After adjusting for smoking quantity (IVW, OR = 1.32, 95%CI 1.01–1.72, *P* = 0.044), drinking frequency (IVW, OR = 1.39, 95%CI:1.09–1.77, P = 0.008), BMI (IVW, OR = 1.22, 95%CI 1.04–1.42, *P* = 0.013), depression (IVW, OR = 1.52, 95%CI 1.12–2.04, *P* = 0.006), and education attainment (IVW, OR = 1.79, 95%CI 1.44–2.23, *P* = 1.29 × 10^−7^) in the discovery set, the genetic susceptibility to GERD is still causally related to RA. After we adjusted smoking quantity (IVW, OR = 1.26, 95%CI 1.03–1.55, *P* = 0.022), drinking frequency (IVW, OR = 1.34, 95%CI 1.14–1.57, *P* = 3.01 × 10^−4^), BMI (IVW, OR = 1.27, 95%CI 1.12–1.43, *P* = 1.37 × 10^−4^), depression (IVW, OR = 1.36, 95%CI 1.12–1.65, *P* = 2.14 × 10^−3^), and education attainment (IVW, OR = 1.49, 95%CI 1.32–1.70, *P* = 5.85 × 10^−10^) on the validation set, the previous conclusions still hold. In sensitivity analyses, MR-Egger regression for multivariate MR analysis did not detect horizontal pleiotropy but there was some degree of heterogeneity, so we used the IVW random-effects method for analysis. Detailed results can be found in the Fig. 5, Table 3 and supplementary Table 11–12.

**Table 3:** Sensitivity analysis of adjusted GERD with risk of RA in multivariable MR analyses.

Exposure	Method	RA (discovery)	RA (validation)
GERD adjusted by BMI	IVW	*P* = 1.31E−02	*P* = 1.37E−04
	MR-Egger	*P* = 1.34E−02	*P* = 1.33E−04
	Heterogeneity	Cochrane’s Q = 1143.21 *P* = 6.48E−62	Cochrane’s Q = 808.85 *P* = 3.78E−24
	Pleiotropy	Intercept = − 0.001 *P* = 7.46E−01	Intercept = 0.002 *P* = 4.35E−01
	F-statistic	8.22	8.17
GERD adjusted by education attainment	IVW	*P* = 1.29E−07	*P* = 5.85E−10
	MR-Egger	*P* = 2.23E−07	*P* = 3.68E−10
	Heterogeneity	Cochrane’s Q = 377.93 *P* = 7.10E−25	Cochrane’s Q = 153.39 *P* = 1.21E−01
	pleiotropy	Intercept = 0.009 *P* = 2.62E−01	Intercept = 0.003 P = 3.81E−01
	F-statistic	15.66	15.7
GERD adjusted by cigarettes per day	IVW	*P* = 4.43E−02	*P* = 2.24E−02
	MR-Egger	*P* = 2.18E−01	*P* = 5.65E−02
	Heterogeneity	Cochrane’s Q = 229.31 *P* = 9.62E−21	Cochrane’s Q = 151.14 *P* = 2.10E−09
	Pleiotropy	Intercept = − 0.008 *P* = 1.96E−01	Intercept = 0.006 *P* = 5.63E−01
	F-statistic	16.22	16.19
GERD adjusted by depression	IVW	*P* = 6.33E−03	*P* = 4.30E−02
	MR-Egger	*P* = 4.42E−01	*P* = 2.43E−01
	Heterogeneity	Cochrane’s Q = 221.17 *P* = 9.93E−20	Cochrane’s Q = 167.37 *P* = 1.46E−09
	Pleiotropy	Intercept = 0.029 *P* = 7.54E−02	Intercept = − 0.001 *P* = 6.82E−01
	F-statistic	5.82	5.75
GERD adjusted by drinks per week	IVW	*P* = 7.61E−03	*P* = 1.13E−02
	MR-Egger	*P* = 2.44E−01	*P* = 1.20E−02
	Heterogeneity	Cochrane’s Q = 261.75 *P* = 6.22E−24	Cochrane’s Q = 188.21 *P* = 6.70E-11
	Pleiotropy	Intercept = − 0.001 *P* = 8.98E−01	Intercept = −0.003 *P* = 3.55E−01
	F-statistic	29.2	29.42

In summary, our results suggest that genetic predisposition to GERD increases the risk of RA, while RA does not have a causal relationship on GERD. This conclusion is supported in different models, and the conclusion that GERD increases the risk of RA remains validated after adjusting for smoking quantity, drinking frequency, BMI, education attainment, and depression.

## Discussion

This study employed a bidirectional TSMR approach on publicly available GWAS data. We demonstrated a positive causal effect of GERD on RA in the European population using a discovery set. The validation set was subsequently used to confirm our findings. The results from the reverse MR analysis indicated a causal effect of RA on GERD was not observed.

Although the availability of new anti-rheumatic drugs has dramatically improved the prognosis and the mortality of RA patients has been declining over the past three decades, the prevalence of the disease has gradually increased^31^. RA is challenging to cure and typically requires lifelong treatment. The damage caused by the disease and the economic burden of treatment continue to be significant global challenges in managing RA^32^. The exact mechanisms underlying the onset of RA remain unclear. Research suggests that aside from genetic factors, environmental elements such as malnutrition, low educational attainment, smoking, occupational exposure to silica, periodontitis, and the microbiome could be associated with the development of RA^33–37^.

Smoking is one of the important factors leading to RA, which not only increases the body's oxidative stress response, but also promotes systemic inflammation and interferes with apoptosis^38^. One meta-analysis reported a 26% increased risk of RA even if the smoker was light (1–10 packs/year) compared with those who had never smoked (RR = 1.26, 95%CI 1.14–1.39)^39^. Low and moderate alcohol consumption has been shown to reduce the risk of RA^40,41^, and one meta-analysis has demonstrated a negative association between alcohol intake and the risk of ACPA-positive RA^42^. It has been reported that there may be a synergistic effect between alcohol and smoking in influencing the risk of developing RA, and that alcohol consumption may lead to a decreased correlation between smoking and the onset of RA^43^. Obesity is considered a risk factor for human health, including but not limited to RA. Results from one meta-analysis suggest that an increase in BMI may be associated with an increased risk of RA (RR = 1.23, 95%CI 1.09–1.39)^14^. Depression is one of the common comorbidities in patients with RA, and epidemiological studies have shown that depression is one of the risk factors for RA. In a study from Taiwan, the risk of RA was significantly higher in depressed people than in non-depressed people (HR = 1.65, 95% CI 1.41–1.77)^44^. Another study of patients in the UK found that the risk of RA increased by 38% in depressed people compared with non-depressed people after adjusting for age, sex, smoking, BMI, comorbidities, and antidepressant use (HR = 1.38, 95% CI 1.31–1.46)^45^. Previous MR studies have also shown that higher educational attainment has a protective effect on RA (OR = 0.37, 95% CI 0.31–0.44)^12^. After performing multivariate MR analyses and correcting for these confounders, we found that genetic susceptibility to GERD was still causally associated with RA.

**Figure 6:**
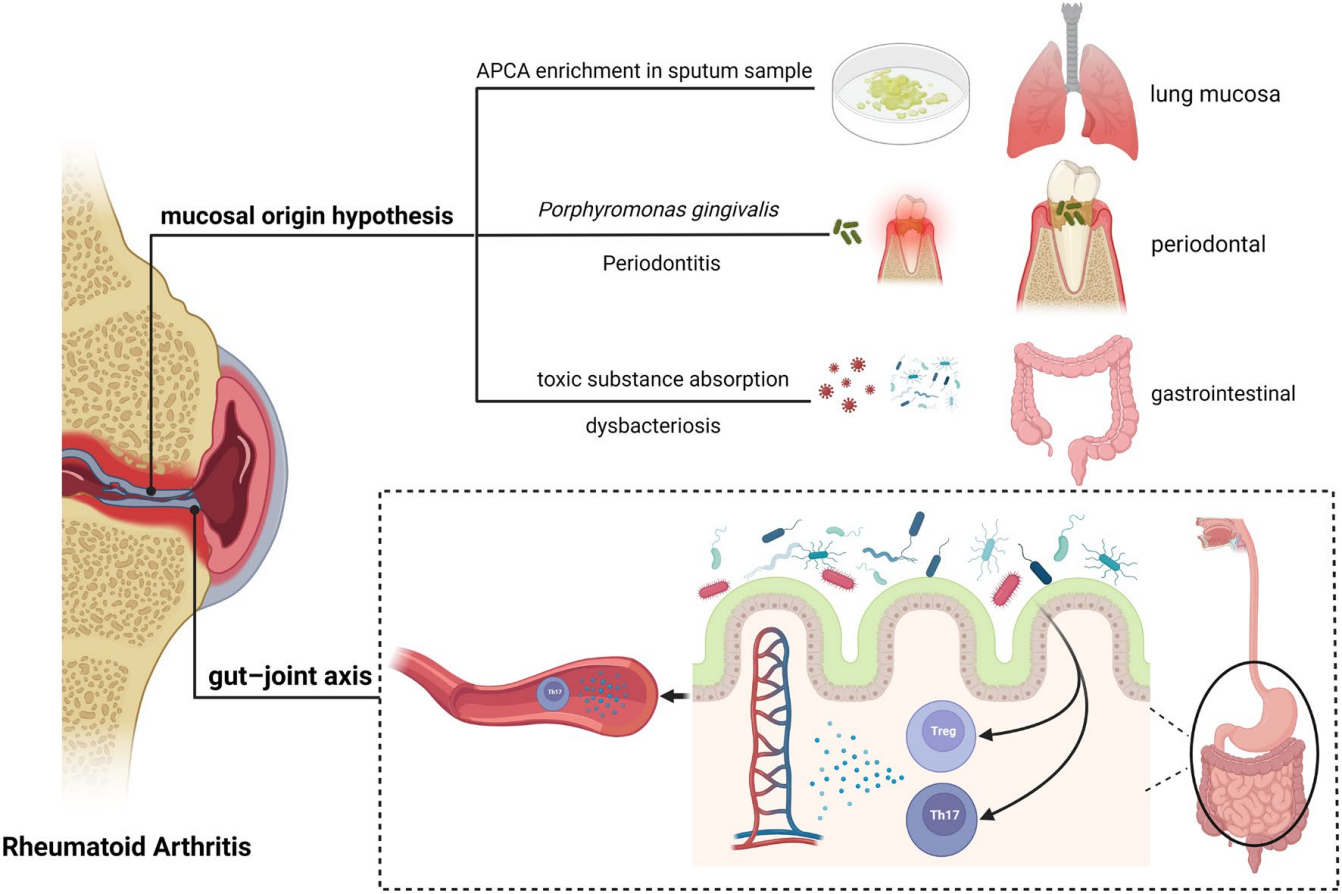
Possible mechanisms leading to RA. Figure illustrates some of the mechanisms underlying mucosal involvement in rheumatoid arthritis, as well as certain gut-joint axis mechanisms mentioned in the text.

However, explaining the causal effect of genetic susceptibility to GERD on RA can be challenging (Fig. 6). One of the hypotheses in the etiology of RA is the "mucosal origin," suggesting that the autoimmune response leading to the development of RA is triggered within the relevant lymphoid tissues in the mucosa of the lungs, oral cavity, and gastrointestinal tract^46^. Factors such as the absorption of toxic substances in the intestines, disruption of gastrointestinal anatomical structures, and alterations in the microbiota can contribute to the formation of synovitis^34^. Studies have indicated that RA-associated autoantibodies can be produced in the pulmonary mucosa and lymph nodes. Local enrichment of anti-citrullinated protein antibodies (ACPA) has been detected in the sputum of early untreated RA patients^47^. Periodontitis and oral microbiota, with representatives like *Porphyromonas gingivalis*, can also contribute to the development of RA^48,49^. GERD, as a form of chronic esophageal damage, is increasingly being considered as a potential contributor to the development of RA, possibly even in its early stages. It's not just regarded as a comorbidity of RA or a complication during RA treatment involving corticosteroids or non-steroidal anti-inflammatory drugs. Abnormal reflux of gastric fluid and contents can damage the esophageal mucosa. Displacement of intestinal bacteria or distal esophageal bacteria may occur, breaking through the mucosal barrier and potentially triggering an immune response. In normal conditions, the esophageal microbiota is primarily composed of Gram-positive bacteria. However, in GERD patients, there is a shift towards an increased presence of Gram-negative bacteria. This includes genera like *Prevotella*, *Haemophilus*, *Neisseria*, *Campylobacter*, and *Clostridium*^50,51^. With the increasing proportion of Gram-negative bacteria, there is also an elevation in lipopolysaccharide content. This can subsequently lead to an upregulation of gene expression through the Toll-like receptor 4 and *NFκB* pathways, resulting in an augmentation of pro-inflammatory cytokine expression^52^. Recent germ-free experiments have substantiated how individual microbial communities’ impact specific immune cell populations, altering the balance between pro-inflammatory cells and regulatory T cells both at mucosal sites and within the bloodstream^53^. Following dysbiosis of the gut microbiota, segmented filamentous bacteria can activate Th17 cells within the lamina propria, leading to a reduced proportion of anti-inflammatory Tregs, exacerbating systemic inflammatory responses, fostering an autoimmune predisposition, and ultimately precipitating arthritis^54–56^. Research conducted by Jose U. Scher indicates a strong association between the presence of *Prevotella copri* in the gut and newly diagnosed, untreated RA^57^. This study also identifies the potential role of this bacterium in the pathogenesis of RA. Coincidentally, GERD patients also exhibit the presence of *Prevotella copri* in the distal esophagus. This bacterium in the distal esophagus may also contribute to the development of RA. A human model study focusing on conditions such as Whipple's disease, which aligns with the gut-joint axis hypothesis, has indicated that *Tropheryma whipplei* can trigger the occurrence of RA in susceptible individuals^58^. It has been reported that this disease not only affects the small intestine but can also involve the esophagus, pharynx, duodenum, colon, and other areas^59^. Furthermore, chronic mucosal inflammation appears to be a significant mechanism in the pathogenesis of RA. Reports suggest TLRs expressed in the esophageal mucosa mediate the interaction between the immune system and the microbiota, which could also be a mechanism driving chronic inflammatory responses^60^.

In addition, non-steroidal anti-inflammatory drugs (NSAIDs) and corticosteroids taken during treatment in RA patients are one of the causes of gastrointestinal complications^61^. In addition, antirheumatic drugs can sometimes cause adverse gastrointestinal events^62^. As a result, the conclusion of some observational studies that there is a link between RA and GERD has been influenced. However, our findings using MR analysis suggest that there is no causal relationship between RA and GERD.

It should be emphasized that RA is a complex autoimmune disease characterized by the interaction of multiple causative factors in its pathogenesis. Therefore, there is a need for more comprehensive and detailed investigation into the mechanisms through which GERD may contribute to the development of RA.

In the reverse MR analysis, we observed that the results from the discovery set indicated an increased risk of GERD associated with RA, while the results from the validation set were entirely opposite. We speculate that this discrepancy might be due to a small overlap in samples between the RA (discovery) and GERD datasets, leading to such outcomes. Since there is no sample overlap between RA (validation) and GERD, such results would not be expected. Theoretically, there is also the possibility of false negatives due to the small size of the validation dataset, but we lean towards the first possibility.

This study still has some limitations. First, due to weak instrumental bias (F-statistic < 10), the causal effect of GERD and RA, which corrected by BMI and depression should be interpreted with caution. In addition, different databases may contribute to the presence of heterogeneity. However, the use of the IVW random-effects method and the absence of horizontal pleiotropy indicates that our results are unlikely to be interfered with by heterogeneity ^62^. Secondly, our GWAS data are derived from European populations, which may limit the generalizability of the MR results when extrapolated to other populations. we did not perform a stratified analysis based on serum ACPA positivity and negativity or gender. Further research in the future could explore this aspect in more detail. Lastly, the credibility of IVs to a certain extent is influenced by the sample size of the GWAS. In the future, larger-scale GWAS data will be necessary to validate the conclusions.

## Conclusion

In summary, our analysis supports a causal relationship between genetic susceptibility to GERD and an increased risk of RA. This finding is crucial for deepening our understanding of the pathogenesis of RA and may offer new insights for the prevention and treatment of RA. It also offers a new perspective on preventing the occurrence of GERD in patients with RA. However, based on the results of the reverse MR analysis using the existing dataset, compelling evidence was not found for RA increasing the risk of developing GERD.

### Supplementary Information

Supplementary Figures.

Supplementary Tables.

## Data Availability

The data requirements mentioned in the article can be found in the article/Supplementary materials. For further details may directly contact the corresponding author through the provided contact information.

## Abbreviations

GERD: Gastroesophageal reflux disease
RA: Rheumatoid arthritis
MR: Mendelian randomization
GWAS: Genome-wide association study
TSMR: Two-sample mendelian randomization
IVs: Instrumental variables
RCTs: Randomized controlled trials
SNPs: Single nucleotide polymorphisms
IVW: Inverse variance weighting
MR-PRESSO: Mendelian randomization pleiotropy residual sum and outlier
ACPA: Anti-citrullinated protein antibodies

## References

[1] Hunt, R. *et al.* World gastroenterology organisation global guidelines: GERD global perspective on gastroesophageal reflux disease. *J. Clin. Gastroenterol.* **51**(6), 467–478 (2017). 28591069 10.1097/MCG.0000000000000854

[2] Schneider, A., Merikhi, A. & Frank, B. B. Autoimmune disorders: gastrointestinal manifestations and endoscopic findings. *Gastrointest. Endosc. Clin. N. Am.* **16**(1), 133–151 (2006). 16546029 10.1016/j.giec.2006.01.013

[3] Chong, V. H. & Wang, C. L. Higher prevalence of gastrointestinal symptoms among patients with rheumatic disorders. *Singap. Med J.* **49**(5), 419–424 (2008). 18465055

[4] Miura, Y., Fukuda, K., Maeda, T. & Kurosaka, M. Gastroesophageal reflux disease in patients with rheumatoid arthritis. *Mod. Rheumatol.* **24**(2), 291–295 (2014). 24252041 10.3109/14397595.2013.843749

[5] Lin, H. C., Xirasagar, S., Lee, C. Z., Huang, C. C. & Chen, C. H. The association between gastro-oesophageal reflux disease and subsequent rheumatoid arthritis occurrence: A nested case-control study from Taiwan. *BMJ Open* **7**(11), e016667 (2017). 29151046 10.1136/bmjopen-2017-016667 PMC5702028

[6] Kim, S. Y., Min, C., Park, B. & Choi, H. G. Bidirectional association between GERD and rheumatoid arthritis: Two longitudinal follow-up studies using a national sample cohort. *Clin. Rheumatol.* **40**(4), 1249–1257 (2021). 32944882 10.1007/s10067-020-05400-0

[7] Qian, Y. *et al.* Genetic predisposition to smoking is associated with risk of rheumatoid arthritis: a Mendelian randomization study. *Arthritis Res. Ther.* **22**(1), 44 (2020). 32143697 10.1186/s13075-020-2134-1 PMC7060545

[8] Wang, J. *et al.* The causal association between alcohol, smoking, coffee consumption, and the risk of arthritis: A meta-analysis of Mendelian randomization studies. *Nutrients* **15**(23), 5009 (2023). 38068867 10.3390/nu15235009 PMC10707754

[9] Yuan, S. *et al.* Smoking, alcohol consumption, and 24 gastrointestinal diseases: Mendelian randomization analysis. *Elife*10.7554/eLife.84051 (2023). 36727839 10.7554/eLife.84051 PMC10017103

[10] Fang, S. *et al.* Assessment of bidirectional relationships between depression and rheumatoid arthritis among adults: A two-sample Mendelian randomization study. *Clin. Rheumatol.* **42**(4), 1039–1046 (2023). 36454344 10.1007/s10067-022-06455-x

[11] Ruan, X. *et al.* Depression and 24 gastrointestinal diseases: a Mendelian randomization study. *Transl. Psychiatry* **13**(1), 146 (2023). 37142593 10.1038/s41398-023-02459-6 PMC10160129

[12] Zhao, S. S., Holmes, M. V., Zheng, J., Sanderson, E. & Carter, A. R. The impact of education inequality on rheumatoid arthritis risk is mediated by smoking and body mass index: Mendelian randomization study. *Rheumatology* **61**(5), 2167–2175 (2022). 34436562 10.1093/rheumatology/keab654 PMC9071527

[13] Zhang, X. *et al.* Association of educational attainment with esophageal cancer, Barrett’s esophagus, and gastroesophageal reflux disease, and the mediating role of modifiable risk factors: A Mendelian randomization study. *Front. Public Health.* **11**, 1022367 (2023). 37056646 10.3389/fpubh.2023.1022367 PMC10086429

[14] Feng, X. *et al.* Body mass index and the risk of rheumatoid arthritis: An updated dose-response meta-analysis. *Biomed. Res. Int.* **2019**, 3579081 (2019). 31355257 10.1155/2019/3579081 PMC6634074

[15] Zafar, S. *et al.* Correlation of endoscopic severity of gastroesophageal reflux disease (GERD) with body mass index (BMI). *J. Coll. Physicians Surg. Pak.* **17**(2), 72–75 (2007). 17288850

[16] Ong, J. S. *et al.* Multitrait genetic association analysis identifies 50 new risk loci for gastro-oesophageal reflux, seven new loci for Barrett’s oesophagus and provides insights into clinical heterogeneity in reflux diagnosis. *Gut* **71**(6), 1053–1061 (2022). 34187846 10.1136/gutjnl-2020-323906 PMC9120377

[17] Ishigaki, K. *et al.* Multi-ancestry genome-wide association analyses identify novel genetic mechanisms in rheumatoid arthritis. *Nat. Genet.* **54**(11), 1640–1651 (2022). 36333501 10.1038/s41588-022-01213-w PMC10165422

[18] Kurki, M. I. *et al.* FinnGen provides genetic insights from a well-phenotyped isolated population. *Nature* **613**(7944), 508–518 (2023). 36653562 10.1038/s41586-022-05473-8 PMC9849126

[19] Saunders, G. R. B. *et al.* Genetic diversity fuels gene discovery for tobacco and alcohol use. *Nature* **612**(7941), 720–724 (2022). 36477530 10.1038/s41586-022-05477-4 PMC9771818

[20] Yengo, L. *et al.* Meta-analysis of genome-wide association studies for height and body mass index in ∼700000 individuals of European ancestry. *Hum. Mol. Genet.* **27**(20), 3641–3649 (2018). 30124842 10.1093/hmg/ddy271 PMC6488973

[21] Lee, J. J. *et al.* Gene discovery and polygenic prediction from a genome-wide association study of educational attainment in 1.1 million individuals. *Nat. Genet.* **50**(8), 1112–21 (2018). 30038396 10.1038/s41588-018-0147-3 PMC6393768

[22] Feng, R. *et al.* Pulmonary embolism and 529 human blood metabolites: genetic correlation and two-sample Mendelian randomization study. *BMC Genom. Data.* **23**(1), 69 (2022). 36038828 10.1186/s12863-022-01082-6 PMC9422150

[23] Kurilshikov, A. *et al.* Large-scale association analyses identify host factors influencing human gut microbiome composition. *Nat. Genet.* **53**(2), 156–165 (2021). 33462485 10.1038/s41588-020-00763-1 PMC8515199

[24] Papadimitriou, N. *et al.* Physical activity and risks of breast and colorectal cancer: a Mendelian randomisation analysis. *Nat. Commun.* **11**(1), 597 (2020). 32001714 10.1038/s41467-020-14389-8 PMC6992637

[25] Burgess, S. & Thompson, S. G. Avoiding bias from weak instruments in Mendelian randomization studies. *Int. J. Epidemiol.* **40**(3), 755–764 (2011). 21414999 10.1093/ije/dyr036

[26] Burgess, S. & Thompson, S. G. Interpreting findings from Mendelian randomization using the MR-Egger method. *Eur. J. Epidemiol.* **32**(5), 377–389 (2017). 28527048 10.1007/s10654-017-0255-x PMC5506233

[27] Verbanck, M., Chen, C. Y., Neale, B. & Do, R. Detection of widespread horizontal pleiotropy in causal relationships inferred from Mendelian randomization between complex traits and diseases. *Nat. Genet.* **50**(5), 693–698 (2018). 29686387 10.1038/s41588-018-0099-7 PMC6083837

[28] Huang, S. *et al.* Physical activity and systemic lupus erythematosus among European populations: A two-sample Mendelian randomization study. *Front Genet.* **12**, 784922 (2021). 35211151 10.3389/fgene.2021.784922 PMC8861300

[29] Brion, M. J., Shakhbazov, K. & Visscher, P. M. Calculating statistical power in Mendelian randomization studies. *Int J Epidemiol.* **42**(5), 1497–1501 (2013). 24159078 10.1093/ije/dyt179 PMC3807619

[30] Faul, F., Erdfelder, E., Lang, A. G. & Buchner, A. G*Power 3: A flexible statistical power analysis program for the social, behavioral, and biomedical sciences. *Behav. Res.Methods* **39**(2), 175–191 (2007). 17695343 10.3758/BF03193146

[31] Finckh, A. *et al.* Global epidemiology of rheumatoid arthritis. *Nat. Rev. Rheumatol.* **18**(10), 591–602 (2022). 36068354 10.1038/s41584-022-00827-y

[32] Joensuu, J. T. *et al.* The cost-effectiveness of biologics for the treatment of rheumatoid arthritis: A systematic review. *PLoS One.* **10**(3), e0119683 (2015). 25781999 10.1371/journal.pone.0119683 PMC4363598

[33] Millar, K. *et al.* Personality, socio-economic status and inflammation: cross-sectional, population-based study. *PLoS One* **8**(3), e58256 (2013). 23516457 10.1371/journal.pone.0058256 PMC3596406

[34] Scher, J. U., Littman, D. R. & Abramson, S. B. Microbiome in inflammatory arthritis and human rheumatic diseases. *Arthritis Rheumatol.* **68**(1), 35–45 (2016). 26331579 10.1002/art.39259 PMC4789258

[35] Li, R. *et al.* Rheumatoid arthritis and periodontal disease: What are the similarities and differences?. *Int. J. Rheum. Dis.* **20**(12), 1887–1901 (2017). 29341486 10.1111/1756-185X.13240

[36] Hu, Y. *et al.* Long-term dietary quality and risk of developing rheumatoid arthritis in women. *Ann. Rheum. Dis.* **76**(8), 1357–1364 (2017). 28137914 10.1136/annrheumdis-2016-210431 PMC5556680

[37] Klareskog, L., Malmström, V., Lundberg, K., Padyukov, L. & Alfredsson, L. Smoking, citrullination and genetic variability in the immunopathogenesis of rheumatoid arthritis. *Semin. Immunol.* **23**(2), 92–98 (2011). 21376627 10.1016/j.smim.2011.01.014

[38] Chang, K. *et al.* Smoking and rheumatoid arthritis. *Int. J. Mol. Sci.* **15**(12), 22279–22295 (2014). 25479074 10.3390/ijms151222279 PMC4284707

[39] Di Giuseppe, D., Discacciati, A., Orsini, N. & Wolk, A. Cigarette smoking and risk of rheumatoid arthritis: A dose-response meta-analysis. *Arthritis Res. Ther.* **16**(2), R61 (2014). 24594022 10.1186/ar4498 PMC4060378

[40] Di Giuseppe, D., Alfredsson, L., Bottai, M., Askling, J. & Wolk, A. Long term alcohol intake and risk of rheumatoid arthritis in women: a population based cohort study. *BMJ* **345**, e4230 (2012). 22782847 10.1136/bmj.e4230 PMC3393782

[41] Lu, B., Solomon, D. H., Costenbader, K. H. & Karlson, E. W. Alcohol consumption and risk of incident rheumatoid arthritis in women: a prospective study. *Arthritis Rheumatol.* **66**(8), 1998–2005 (2014). 24729427 10.1002/art.38634 PMC4116451

[42] Scott, I. C. *et al.* The protective effect of alcohol on developing rheumatoid arthritis: A systematic review and meta-analysis. *Rheumatology* **52**(5), 856–867 (2013). 23287363 10.1093/rheumatology/kes376

[43] Hedenstierna, L. *et al.* Effects of alcohol consumption and smoking on risk for RA: Results from a Swedish prospective cohort study. *RMD Open*10.1136/rmdopen-2020-001379 (2021). 33414179 10.1136/rmdopen-2020-001379 PMC7797247

[44] Lu, M. C. *et al.* Bidirectional associations between rheumatoid arthritis and depression: A nationwide longitudinal study. *Sci. Rep.* **6**, 20647 (2016). 26857028 10.1038/srep20647 PMC4746638

[45] Vallerand, I. A. *et al.* Depression as a risk factor for the development of rheumatoid arthritis: a population-based cohort study. *RMD Open* **4**(2), e000670 (2018). 30018804 10.1136/rmdopen-2018-000670 PMC6045711

[46] Holers, V. M. *et al.* Rheumatoid arthritis and the mucosal origins hypothesis: Protection turns to destruction. *Nat. Rev. Rheumatol.* **14**(9), 542–557 (2018). 30111803 10.1038/s41584-018-0070-0 PMC6704378

[47] Willis, V. C. *et al.* Sputum autoantibodies in patients with established rheumatoid arthritis and subjects at risk of future clinically apparent disease. *Arthritis Rheum.* **65**(10), 2545–2554 (2013). 23817979 10.1002/art.38066 PMC4066465

[48] Zhou, N. *et al.* Porphyromonas gingivalis induces periodontitis, causes immune imbalance, and promotes rheumatoid arthritis. *J. Leukoc. Biol.* **110**(3), 461–473 (2021). 34057740 10.1002/JLB.3MA0121-045R

[49] de Molon, R. S., Rossa, C. Jr., Thurlings, R. M., Cirelli, J. A. & Koenders, M. I. Linkage of periodontitis and rheumatoid arthritis: Current evidence and potential biological interactions. *Int. J. Mol. Sci.* **20**(18), 4541 (2019). 31540277 10.3390/ijms20184541 PMC6769683

[50] Yang, L. *et al.* Inflammation and intestinal metaplasia of the distal esophagus are associated with alterations in the microbiome. *Gastroenterology* **137**(2), 588–597 (2009). 19394334 10.1053/j.gastro.2009.04.046 PMC2963147

[51] Blackett, K. L. *et al.* Oesophageal bacterial biofilm changes in gastro-oesophageal reflux disease, Barrett’s and oesophageal carcinoma: association or causality?. *Aliment Pharmacol. Ther.* **37**(11), 1084–1092 (2013). 23600758 10.1111/apt.12317

[52] Yang, L., Francois, F. & Pei, Z. Molecular pathways: pathogenesis and clinical implications of microbiome alteration in esophagitis and Barrett esophagus. *Clin. Cancer Res.* **18**(8), 2138–2144 (2012). 22344232 10.1158/1078-0432.CCR-11-0934 PMC3725293

[53] Catrina, A. I., Deane, K. D. & Scher, J. U. Gene, environment, microbiome and mucosal immune tolerance in rheumatoid arthritis. *Rheumatology* **55**(3), 391–402 (2016). 25539828 10.1093/rheumatology/keu469 PMC4746430

[54] Wu, H. J. *et al.* Gut-residing segmented filamentous bacteria drive autoimmune arthritis via T helper 17 cells. *Immunity.* **32**(6), 815–827 (2010). 20620945 10.1016/j.immuni.2010.06.001 PMC2904693

[55] Wang, Y. *et al.* Induction of intestinal Th17 Cells by flagellins from segmented filamentous bacteria. *Front. Immunol.* **10**, 2750 (2019). 31824516 10.3389/fimmu.2019.02750 PMC6883716

[56] Zanin-Zhorov, A. *et al.* Protein kinase C-theta mediates negative feedback on regulatory T cell function. *Science.* **328**(5976), 372–376 (2010). 20339032 10.1126/science.1186068 PMC2905626

[57] Scher, J. U. *et al.* Expansion of intestinal *Prevotella copri* correlates with enhanced susceptibility to arthritis. *Elife* **2**, e01202 (2013). 24192039 10.7554/eLife.01202 PMC3816614

[58] Moos, V. & Schneider, T. Changing paradigms in Whipple’s disease and infection with *Tropheryma whipplei*. *Eur. J. Clin. Microbiol. Infect. Dis.* **30**(10), 1151–1158 (2011). 21461659 10.1007/s10096-011-1209-y

[59] Marcial, M. A. & Villafaña, M. Whipple’s disease with esophageal and colonic involvement: Endoscopic and histopathologic findings. *Gastrointest. Endosc.* **46**(3), 263–266 (1997). 9378216 10.1016/S0016-5107(97)70098-1

[60] Baghdadi, J., Chaudhary, N., Pei, Z. & Yang, L. Microbiome, innate immunity, and esophageal adenocarcinoma. *Clin. Lab. Med.* **34**(4), 721–732 (2014). 25439272 10.1016/j.cll.2014.08.001 PMC4254553

[61] Smolen, J. S., Aletaha, D. & McInnes, I. B. Rheumatoid arthritis. *Lancet* **388**(10055), 2023–2038 (2016). 27156434 10.1016/S0140-6736(16)30173-8

[62] Singh, G. *et al.* Toxicity profiles of disease modifying antirheumatic drugs in rheumatoid arthritis. *J. Rheumatol.* **18**(2), 188–194 (1991). 1673721

