# Supplementary material for: Gastroesophageal reflux disease increases the risk of rheumatoid arthritis: a bidirectional two-sample Mendelian randomization study
Source: Sci Rep. 2024 Aug 1;14:17796. doi: 10.1038/s41598-024-64966-w (PMC11294333; doi:10.1038/s41598-024-64966-w)
Supplement: Supplementary file 1 — Supplementary Figures. [file 41598_2024_64966_MOESM1_ESM.docx]

**Supplementary Materials: Leave-one-out plots and funnel plots for the forward and reverse MR analyses.**


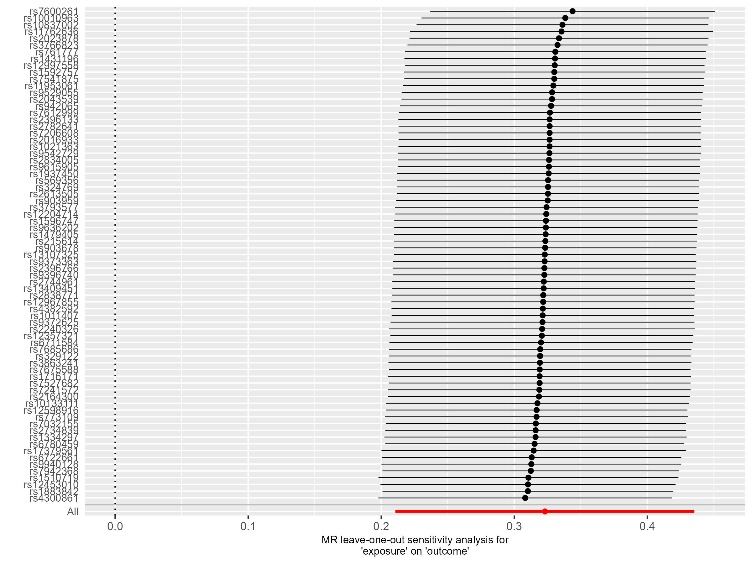

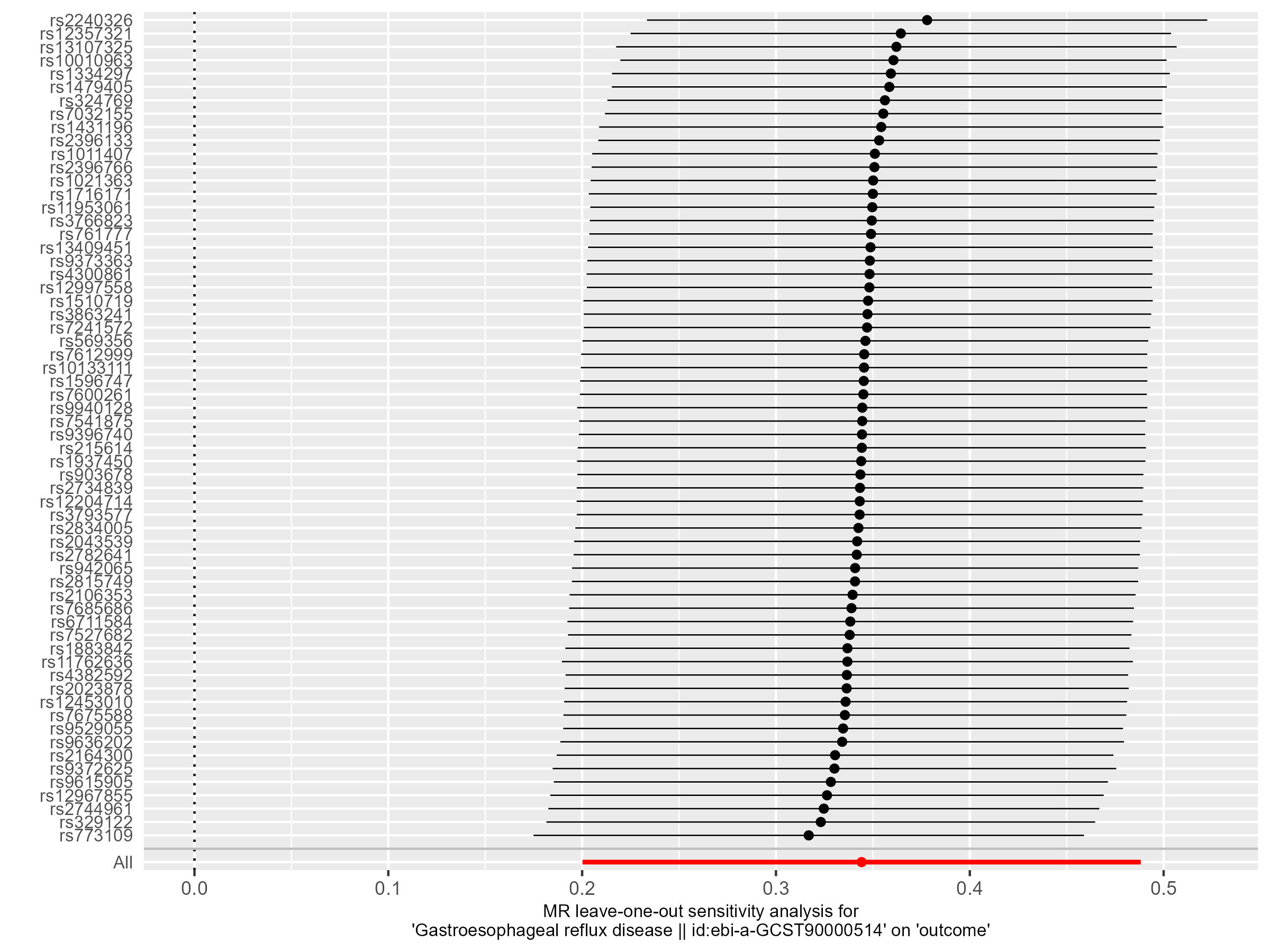


**Figue1.** The leave-one-out plot of MR analysis of GERD as exposure. On the left is GERD on RA discovery, on the right is GERD on RA validation. GERD, Gastroesophageal reflux disease; RA, rheumatoid arthritis.


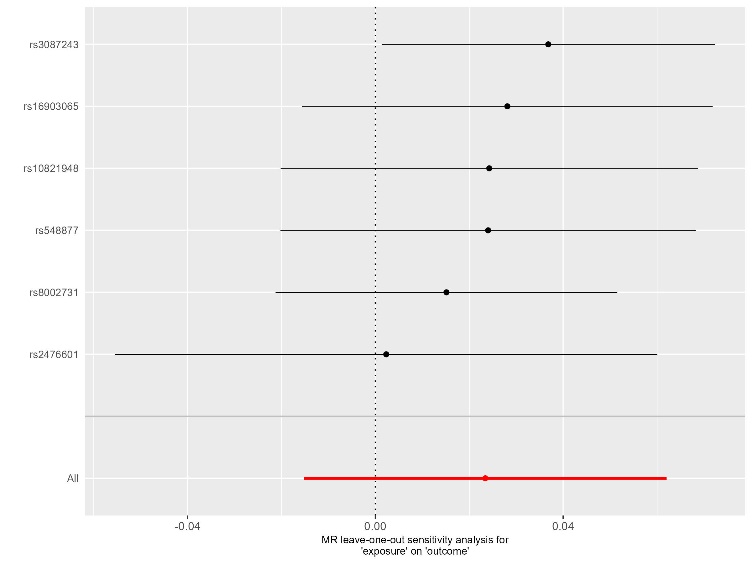

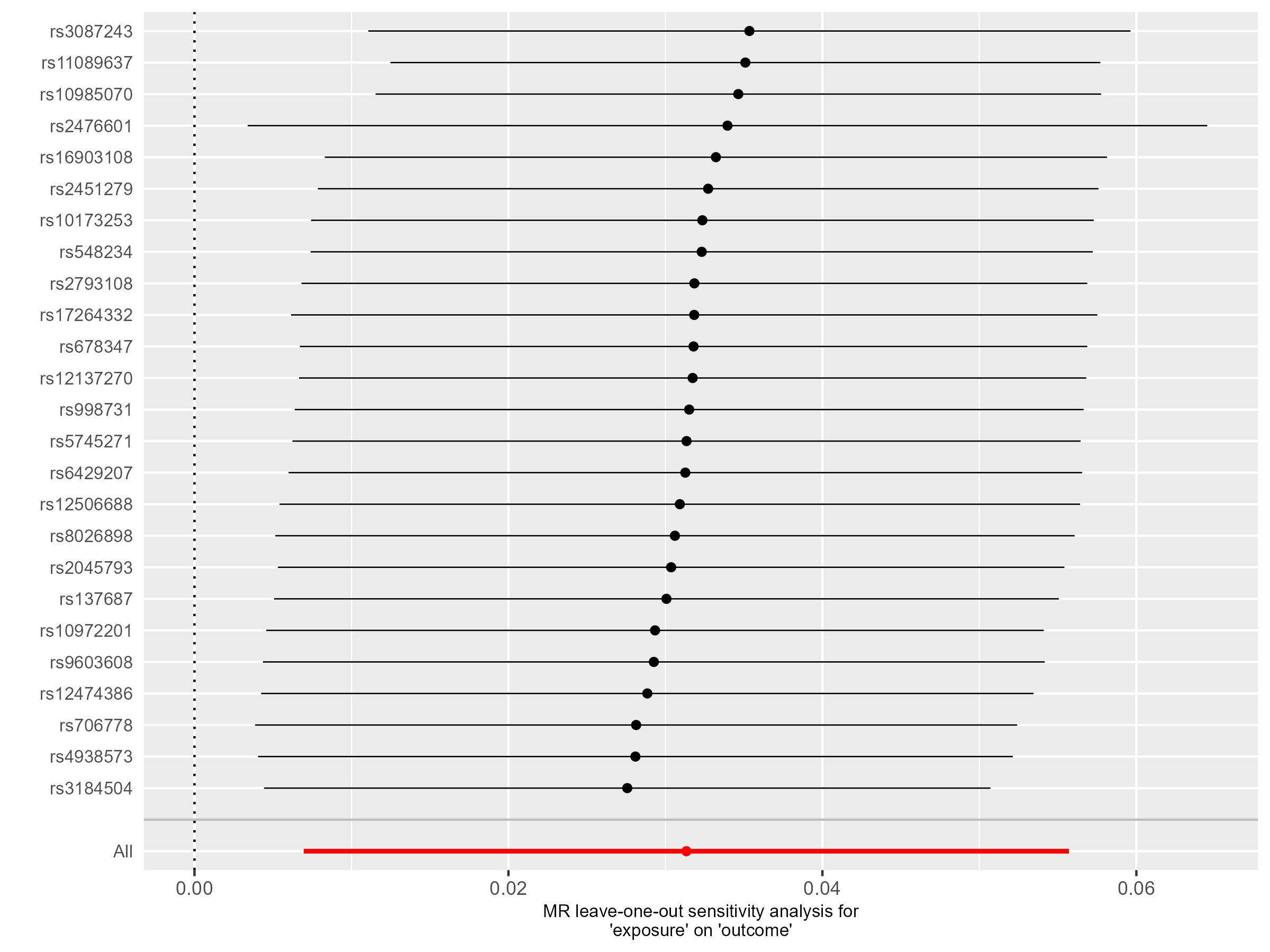


**Figue2.** The leave-one-out plot results of MR analysis of RA as exposure. On the left is RA discovery on GERD, on the right is RA validation on GERD.


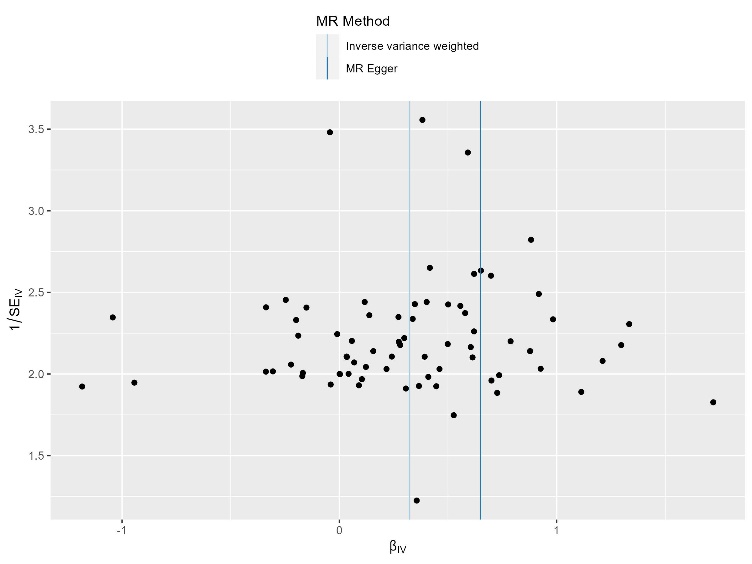

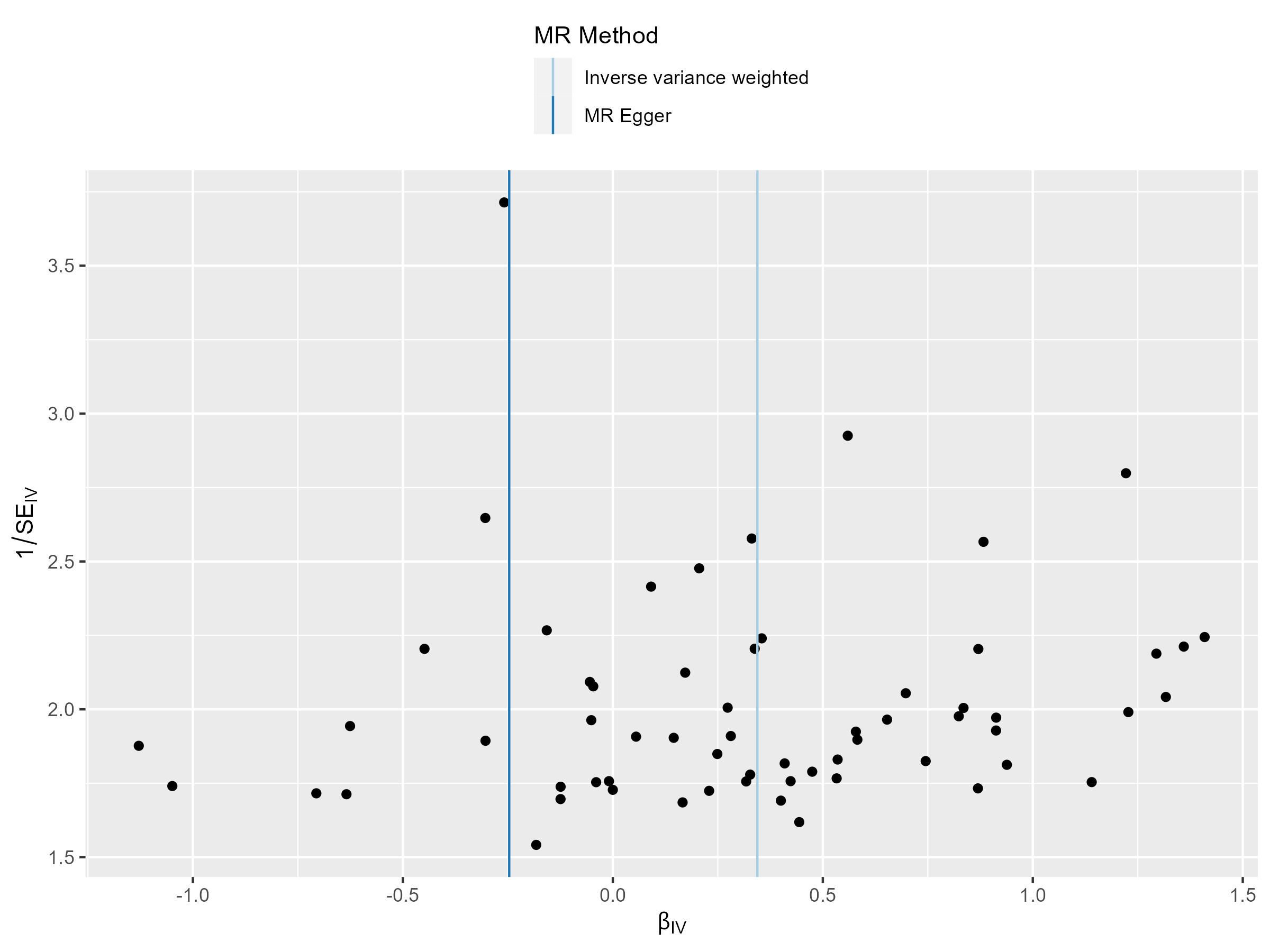


**Figue3.** The funnel plot results of MR analysis of GERD as exposure. On the left is GERD on RA discovery, on the right is GERD on RA validation.


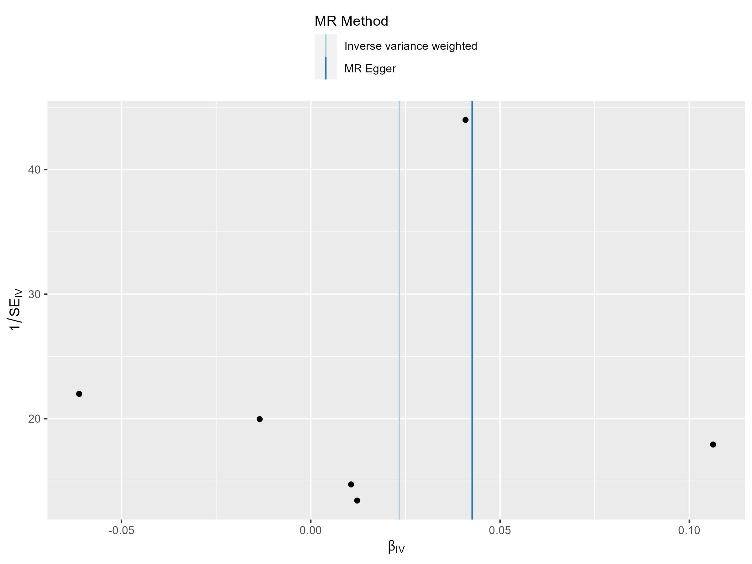

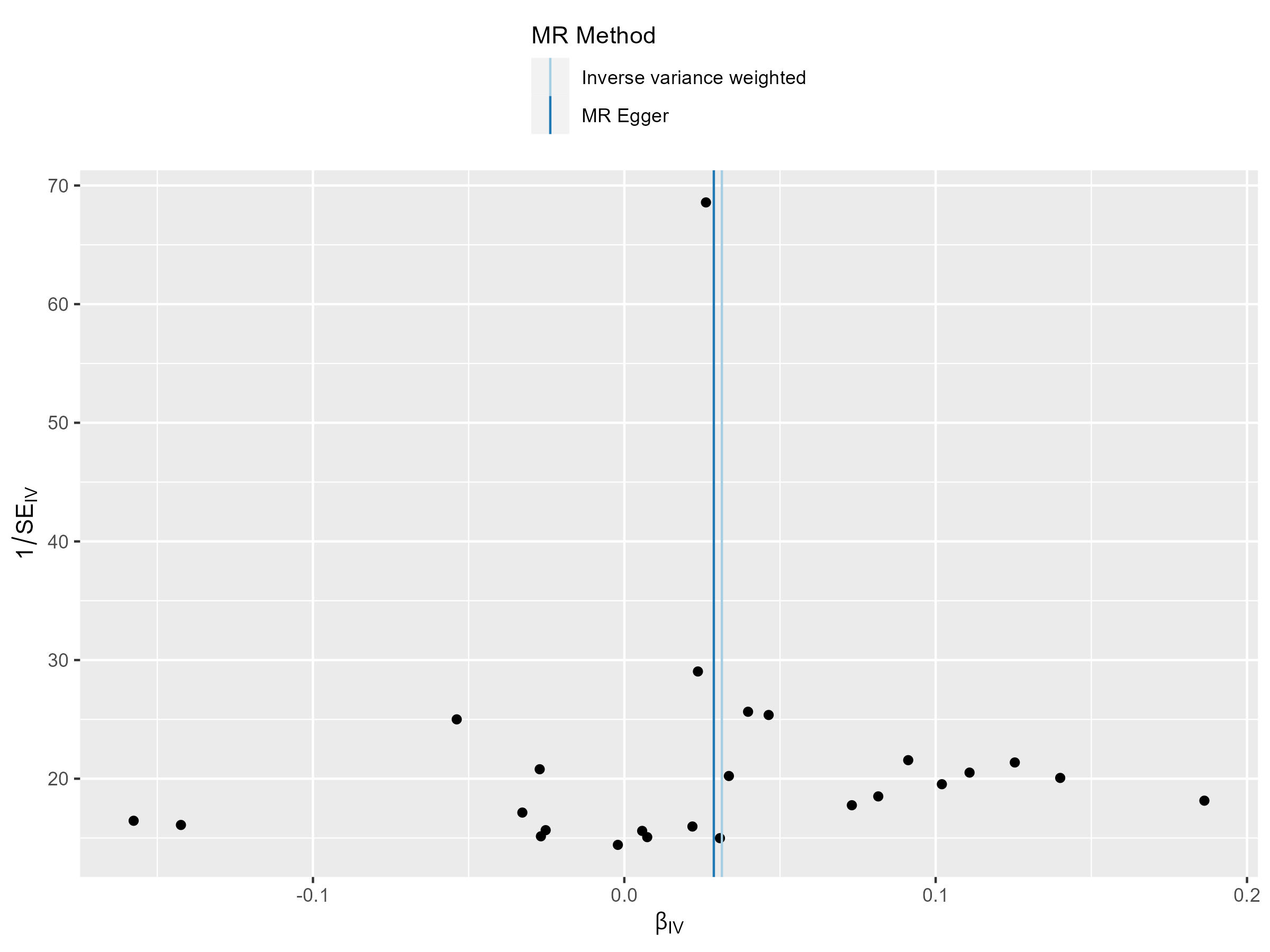


**Figue4.** The funnel plot results of MR analysis of RA as exposure. On the left is RA discovery on GERD, on the right is RA validation on GERD.
